# Where to Go from Here? An Exploratory Meta-Analysis of the Most Promising Approaches to Depression Prevention Programs for Children and Adolescents

**DOI:** 10.3390/ijerph120504758

**Published:** 2015-04-30

**Authors:** Sarah E. Hetrick, Georgina R. Cox, Sally N. Merry

**Affiliations:** 1 Orygen, The National Centre of Excellence in Youth Mental Health, 35 Poplar Rd, Parkville, VIC, 3052, Australia; E-Mail: gcox@unimelb.edu.au; 2 The Werry Centre for Child and Adolescent Mental Health, Department of Psychological Medicine, School of Medicine, Faculty of Medical and Health Sciences, University of Auckland, Private Bag 92019, Auckland 1142, New Zealand; E-Mail: s.merry@auckland.ac.nz

**Keywords:** prevention, depressive disorder, evidence-based practice, children, adolescent, meta-analysis

## Abstract

*Objective*: To examine the overall effect of individual depression prevention programs on future likelihood of depressive disorder and reduction in depressive symptoms. In addition, we have investigated whether Cognitive Behavioural Therapy (CBT), Interpersonal Therapy (IPT) and other therapeutic techniques may modify this effectiveness. *Methods*: This study is based on and includes the trial data from meta-analyses conducted in the Cochrane systematic review of depression prevention programs for children and adolescents by Merry *et al*. (2011). All trials were published or unpublished English language randomized controlled trials (RCTs) or cluster RCTs of any psychological or educational intervention compared to no intervention to prevent depression in children and adolescents aged 5–19 years. *Results*: There is some evidence that the therapeutic approach used in prevention programs modifies the overall effect. CBT is the most studied type of intervention for depression prevention, and there is some evidence of its effectiveness in reducing the risk of developing a depressive disorder, particularly in targeted populations. Fewer studies employed IPT, however this approach appears promising. To our knowledge, this is the first study to have explored how differences in the approach taken in the prevention programs modify the overall treatment effects of prevention programs for children and adolescents. *Conclusions*: More research is needed to identify the specific components of CBT that are most effective or indeed if there are other approaches that are more effective in reducing the risk of future depressive episodes. It is imperative that prevention programs are suitable for large scale roll-out, and that emerging popular modes of delivery, such as online dissemination continue to be rigorously tested.

## 1. Introduction

Depressive disorder is a common mental health problem for young people throughout the world. Meta-analyses suggest the prevalence of depressive disorder in children under 13 to be at 2.8%, rising to 5.7% in adolescents [1] and adolescence and young adulthood is the peak period for the emergence of new cases of depression [2]. As relapse rates for depressive disorder are high [3], there are a variety of potential negative, long term outcomes associated with it, including difficulties with interpersonal relationships, poor vocational attainment and achievement [4,5] and increased risks of self-harm and suicide [6]. Consequently, intervening in childhood and adolescence to prevent the onset of depressive disorder is likely to be the most effective strategy to prevent the negative outcomes associated with the disorder. 

Over the past 30 years, numerous programs have been developed with the aim of either preventing depressive disorder, or reducing already present depressive symptoms. Some interventions are delivered to all participants (a universal approach), whilst others target those at risk of developing depression, for example, those with a depressed caregiver, or those who have some depressive symptoms, but are not yet clinically depressed (a targeted approach). Evidence derived from systematic reviews and meta-analyses of depression prevention programs suggest that the outcomes with regard to depression prevention are promising, but highlight that in most cases this is only in terms of reducing levels of depressive symptoms, and only in some cases, episodes of clinically significant depression [7,8,9,10,11]. A recent Cochrane Review undertaken by Merry *et al*. [12] included sixty eight Randomized Controlled Trials (RCTs) of depression prevention programs for children and adolescents. The findings indicate a small but significant effect size suggesting small but positive effects in reducing depressive symptoms, and future clinically significant episodes of depression up to 12 months after the intervention is delivered. There are some differences in outcomes depending on whether the interventions were targeted or universal: consistent with previous analyses [7,8], targeted programs showed the largest effects, which were maintained to 12 months; whereas universal programs only showed this effect 3 to 9 months after the intervention was delivered. Findings were less robust when an intervention was compared with an active control and there were weaknesses across the studies, including the lack of rigorous measures of depressive disorders, rather than depressive symptoms. It has also been difficult to show effect when effectiveness rather than efficacy studies are conducted [13,14].

Recently there has been interest in the effectiveness of specific depression prevention programs. For example, a meta-analytic review by Brunwasser *et al*. [15] investigated the effectiveness of the Penn Resiliency Program (PRP), one of the most widely disseminated group based prevention programs. The results of the review suggest that overall PRP produced small positive effects in reducing depressive symptoms in youth at post intervention and up to 1-year follow-up, compared with those who received no intervention. However, the PRP did not significantly impact on rates of depressive disorder, and only three trials measured rates of depressive disorder diagnosis at follow-up. Further, the analysis suggested that while targeted programs produced small positive effects up to 12 months after PRP was delivered, universal programs did not have the same impact on depression symptoms post intervention or at 6 to 8 months post intervention, but only at 12 months post intervention. To our knowledge no other reviews have sought to understand the effectiveness of particular prevention programs for depression in children and adolescents.

The depression prevention programs used thus far are predominantly based on the principles of Cognitive Behavioural Therapy (CBT), Interpersonal Psychotherapy (IPT), problem solving, and/or psycho education. The sixty-eight RCTs included in the review by Merry *et al*. [12] have a great deal of overlap in terms of methods (often group programs delivered in schools) and while findings are promising, they are difficult to replicate and to implement. If we are to make progress it is important that we understand the therapeutic approach taken in these programs in order to develop a public health intervention that can be used reliably across regions or countries to prevent depression. 

In this systematic review, we present a secondary analyses of the data set used in the meta-analysis undertaken by Merry *et al*. [12]. Our aim is to examine in exploratory secondary analyses how CBT, IPT and other therapeutic approaches as well as how individual depression prevention programs (e.g., such as the Penn Resiliency Program and Coping with Stress), modify the overall prevention effect exerted by depression prevention programs. 

## 2. Materials and Methods

**Figure 1:**
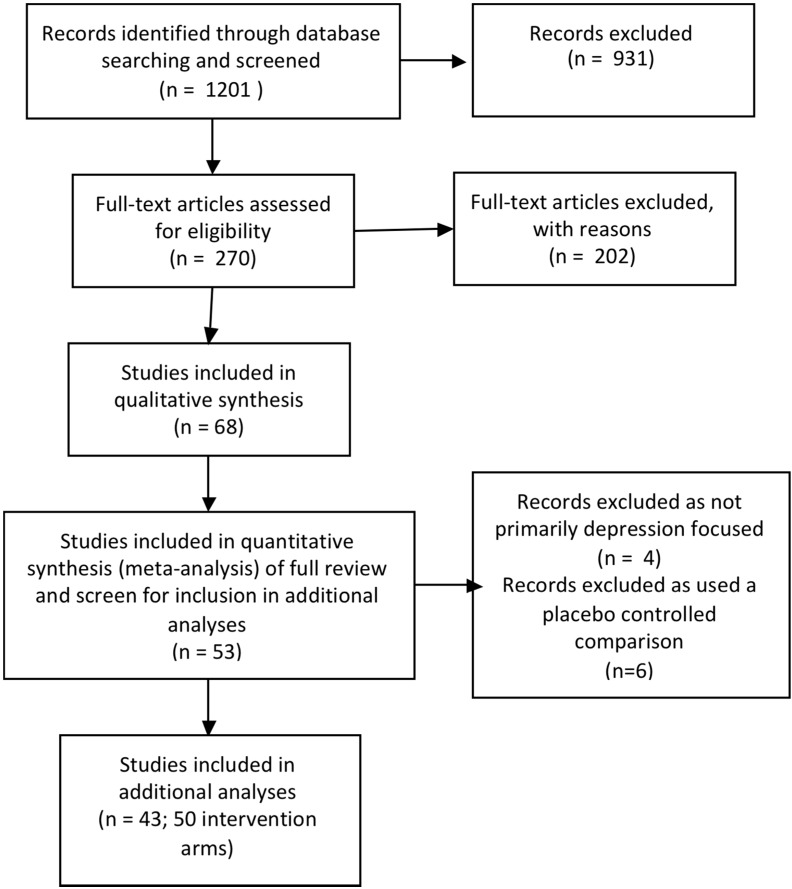
Flow diagram of trials.

This paper is based on a Cochrane systematic review of depression prevention programs for children and adolescents [12] and focuses on additional exploratory analyses of the dataset from this review. The full Cochrane review contains 68 trials. In this study we have only included those trials that provided data for the meta-analyses for post intervention, 3 to 9 month and 12 month follow-up. As our main aim was to explore how various characteristics of interventions tested in prevention trials modified treatment effects, we sought to reduce heterogeneity between the trials and as a result, excluded the following: trials that compared an intervention to a placebo group (where non specific factors are controlled but no active treatment ingredients are included) and those trials where the primary aim or target was not depression prevention. (see Figure 1 for flow diagram). Therefore, in all there are 43 trials with 50 intervention arms included in this secondary analysis. The Cochrane Collaboration systematic review methodology was used as outlined in the Cochrane Handbook for Systematic Reviews of Interventions [16] and there is a full description of the methods in Merry *et al*., 2011 [12]. 

In this paper, we have investigated the impact of particular therapeutic approaches and more specifically of the CBT based programs on these overall results. 

### 2.1. Search Strategy

Briefly, the Cochrane Depression, Anxiety and Neurosis Group trials registers were searched from inception to August 2010 (details can be obtained from authors; details of CCDAN’s generic search strategies can be found in the ‘Specialized Register’ section of the Cochrane Depression, Anxiety and Neurosis Group’s module text but include weekly searches of MEDLINE, EMBASE and PsycINFO; quarterly searches of the Cochrane Central Register of Controlled Trials (CENTRAL) and review specific searches of additional databases as well as reports of trials via the World Health Organisation’s trials portal (ICTRP) (http://apps.who.int/trialsearch/), drug companies, hand-searching of key journals, conference proceedings and other (non-Cochrane) systematic reviews and meta-analyses). In addition to this search, hand searching of Conference abstracts, 1994, 1996, and 1998–2001, for the American Academy of Child and Adolescent Psychiatry were searched, and key investigators in the field were contacted to locate unpublished studies.

### 2.2. Study Selection

All studies were published or unpublished English language RCTs (including cluster RCTs) of any psychological or educational intervention compared to no intervention to prevent depression in children and adolescents (aged 5 to 19 years). Participants did not meet the criteria for a clinical diagnosis of depressive illness, although they may have had sub-clinical symptoms of disorder. Studies that included participants with a history of depression were included if the intervention was aimed at the prevention of depressive disorder or depressive symptoms, and where the participants were not being currently treated for depression.

### 2.3. Assessment of Risk of Bias

Trials were assessed for risk of bias according to the *Cochrane Handbook for Systematic Reviews of Interventions* and more specifically, the “Risk of Bias” tool it recommends [17]. Specifically, we examined each study for randomised sequence generation method, allocation concealment, blinding of participants and assessors, the methods of addressing incomplete outcome data, potential selective reporting, and any other possible bias that might affect the outcome of the study. All assessments of the quality of trials were performed independently by two authors with discrepancies were resolved by a third author.

### 2.4. Statistical Analyses

We extracted the number of people meeting criteria for a depressive disorder as the primary outcome for this review. This was based on either: 1. A standardized diagnostic tool yielding a diagnosis or; 2. A pre-determined (by the trial author) clinical cut-off on a depression symptom measure, including the Beck Depression Inventory (scores over 30), the Children’s Depression Inventory (cut points ranged from 12 to 20), the Centre for Epidemiological Studies-Depression (score equal to or over 24) and the Children’s Depression Rating Scale-Revised (T scores over 65).

These data were pooled using the Risk Difference. We adjusted for the study population numbers to take into account the effect of clustering in cluster RCTs using interclass correlations (ICC) obtained from authors or, if this was not possible, using an ICC estimate of 0.02 (the average of those obtained). For our secondary outcome, depressive symptoms, because different scales were used to measure the same outcomes, the standardized mean difference (SMD) was calculated. 

For all meta-analyses we used the random effects model with a 95% confidence interval. Random effects are, in general, more conservative than fixed-effects models because they take heterogeneity among studies into account [16].

### 2.5. Subgroup Analysis

Subgroup analysis allows investigation of whether the intervention effects vary with different intervention characteristics. Analysis of subgroups by the therapeutic approach used in the intervention was undertaken according to the following categories: CBT (including online CBT), IPT, and other. We categorized trials as ‘CBT’ if cognitive restructuring was described. 

Further subgroup analysis was undertaken of those studies that were categorized as CBT according to the named program that was tested, or approach if there was no specific program name including: the Penn Resiliency Program (PRP), Ease of Handling Social Aspects in Everyday Life Training (LISA-T), Resourceful Adolescent Program (RAP), the stress inoculation approach, the Positive Thinking Program, FRIENDS, The Blues Program, Coping with Stress, Confident Kids, Learn Young Learn Fair, Teaching Kids to Cope, MoodGYM, Problem Solving for Life, with the remainder classified as “unspecified”.

Two authors (SH and GC) independently read the descriptions of the interventions of each included study (see Table 1) and coded them according to the type of intervention and the type of CBT program. Discrepancies in this coding were resolved by a third author (SM). 

To investigate treatment effects in these different subgroups, the overlap of the confidence intervals of the summary estimates was considered. In addition, significant differences between subgroups were explored following the method of Borenstein et al [18] as implemented in RevMan 5.1 [19]. The procedure involves undertaking a standard test for heterogeneity across subgroup results rather than across individual study results.

**Table 1:** Characteristics and coding of nature of interventions tested in included studies.

Study Name	Description	Therapeutic Approach	Specific CBT Program Name
Arnarson 2009 [20]	Based on a number of previous programs including the Coping with Depression and its derivative Coping with Stress program. The focus was on the development of adaptive coping skills to enhance self-esteem and well-being. Stated it incorporated principles of interpersonal therapy, problem solving, behavioural, and cognitive models (pg 581).	CBT & IPT	Unspecified
Balle 2009 [21]	Based on the FRIENDS program; includes education about anxiety, cognitive restructuring, emotional regulation techniques (activation control strategies, controlled breathing, relaxation and cognitive distraction), and gradual exposure to feared situations.	CBT	FRIENDS
Berry 2009 [22]	The Confident Kids program focuses on anxiety and included psychoeducation, cognitive restructuring and graded exposure. Also included education about bullying, coping strategies for bullying situations and sessions on social skill and self esteem enhancement.	CBT	Confident Kids
Barnet 2007 [23]	Trained home visitors provided parenting curriculum (child development, parenting skills, appropriate health care use), encouraged contraceptive use, connected adolescent with primary care, school continuation, provided mentoring and case management, sought to identify depression, partner abuse and school drop out and follow-up of these issues.	Other	
Bond 2004 [24]	The Gatehouse project is a school health promotion program with both individual and ‘whole school’ focused components. The individual component focused on teaching students to identify difficult/conflicting emotional responses to common social situations and develop strategies for responding. The whole school component included a school based adolescent health team as well as interventions to address identified risk and protective factors in the schools social and learning environment.	Other	
Cabiya 2008 [25]	Primarily social problem solving that included teaching adolescents how to understand social cues, how to make accurate interpretations of these cues; how to generate a variety of solutions to a problem they perceive in the social setting; how to decide which solution to enact and how to enact the chosen solution.	CBT	Unspecified
Calear 2009 [26]	MoodGYM is an online CBT program that includes cognitive restructuring, interpersonal skills, relaxation and problem solving. It is fully automated and self-directed.	CBT - online	MoodGYM
Cardemil 2002 a [27]-African American	The Penn Resiliency Program (PRP) includes cognitive restructuring, relaxation and emotion regulation, assertiveness, coping skills, negotiation, social skills, creative and social problem solving, and decision-making.	CBT	PRP
Cardemil 2002 b [27]-Latina	Penn Resiliency Program (PRP)	CBT	PRP
Chaplin a 2006 [28]-girls only	Penn Resiliency Program (PRP)	CBT	PRP
Chaplin b2006 [28]-co-ed	Penn Resiliency Program (PRP)	CBT	PRP
Clarke 1993 [29]	Behavioural skill training intervention that focused on increasing daily rates of pleasant activities.	CBT	Unspecified
Clarke 1995 [30]	The Adolescent Coping with Stress program teaches cognitive restructuring and problem solving skills. The course was based on the “Adolescent Coping with Depression Course” (Clarke *et al*. 1990). Specifically it teaches adolescents to (a) monitor daily moods; (b) identify activating events; (c) discover, challenge, realistically evaluate, and revise negative beliefs; (d) recognize the connections among activating events, beliefs, and consequences (e.g., affect and behaviours); and (e) problem solve and cope with stressful events.	CBT	Coping with Stress
Clarke 2001 [31]	Adolescent Coping with Stress program	CBT	Coping with Stress
Garber 2009 [32]	Adolescent Coping with Stress program with behavioural activation, relaxation and assertiveness training as part of the continuation phase	CBT	Coping with Stress
Gillham 1995 [33]	Included cognitive restructuring, and social problem solving. The social problem solving component focused on conduct problems and interpersonal problems often associated with depression and included teaching children to thinking about their goals before acting, generating a list of possible solutions for problems and making decisions about which solution to enact based on pro’s and con’s of each. They were also taught skills to help cope with parental conflict, and behavioural techniques to enhance assertiveness, negotiation and relaxation.	CBT	PRP
Gillham & Reivich 2006 [34]	Penn Resiliency Program (PRP) with parent component included based on the theory that children learn interpretive and coping styles from their parents, and that helping to prevent or reduce depression in parents interrupts transmission from parents to children.	CBT	PRP
Gillham & Hamilton 2006 [35]	Penn Resiliency Program (PRP)	CBT	PRP
Gillham 2007 [36]	Penn Resiliency Program (PRP)	CBT	PRP
Hains 1990 [37]	Based on cognitive-behavioural stress-inoculation training model developed by Meichenbaum (1985). Included cognitive restructuring around common self defeating cognitions lead to stress and anger.	CBT	Stress Inoculation
Hains 1992 [38]	One group received stress inoculation as in Hains 1990; the second group received anxiety management training following the Suinn 1986 manual that includes learning how to recognise cues that signal the onset of anxiety and the use of relaxation skills to relieve anxiety.	CBT	Stress Inoculation
Horowitz a 2007 [39]	Derived from the Adolescent Coping with Stress program	CBT	Coping with Stress
Horowitz b 2007 [39]	Derived from the IPT–AST (Young & Mufson, 2003) course. IPT-AST includes two individual sessions and 8 group sessions delivering psychoeducation about the relationship between interpersonal difficulties and depression and skill building including communication and interpersonal strategies related to three interpersonal problem areas: interpersonal role disputes, role transitions, and interpersonal deficits.	IPT	IPT-AST
Hyun 2005 [40]	The program integrated cognitive and behavioral components. The cognitive components included identifying reasons for running away from home, identifying high-risk situations including negative emotional states, cognitive distortions and dysfunctional coping strategies, and behavioral components included developing coping strategies such as pleasant activities and relaxation and planning for future life.	CBT	Unspecified
Kraag 2009 [41]	The Learn Young, Learn Fair program addressed stress, stress awareness and coping skills.	CBT	Learn Young, Learn Fair
Lock 2003 [42]	The FRIENDS program (Barrett 2000) was originally based on the Coping Cat (Kendall, 1990) and Coping Koala (Barrett, 1998) programs. It included education about anxiety, cognitive restructuring, emotional regulation techniques (activation control strategies, controlled breathing, relaxation and cognitive distraction), and gradual exposure to feared situations (including interoceptive exposure).	CBT	FRIENDS
Lowry-Webster 2001 [43]	FRIENDS program.	CBT	FRIENDS
Pössel 2004 [44]	The Ease of Handling Social Aspects in Everyday Life-Training (LISA-T) program is based on cognitive behavioural therapy and includes cognitive restructuring as well as a social focus with models of assertiveness and social competence training which targets students ability to develop and maintain social contacts.	CBT	LISA-T
Pössel 2008 [45]	LISA-T	CBT	LISA-T
Puskar 2003 [46]	The Teaching Kids to Cope program is aimed to teach skills that help young people cope with problems and stress. It includes cognitive restructuring but has more emphasis on behavioural skill building including social skills training, assertiveness training, conflict resolution and relaxation. It uniquely includes bibliotherapy, role-playing, and group exercises such as ‘trust-fall’, buddy assignments, and role playing situations from school as well as art activities.	CBT	Teaching Kids to Cope
Quayle 2001 [47]	Adapted PRP and called the Optimism and Life Skills Program	CBT	PRP
Rivet-Duval 2010 [48]	The Resourceful Adolescent Program (RAP) integrates elements of cognitive behavioural therapy (CBT) and interpersonal therapy. It includes behavioural activation with a focus on activities that increase self-esteem, cognitive restructuring, relaxation techniques, problem solving and conflict resolution.	CBT & IPT	RAP
Roberts 2003 [49]	Penn Resiliency Program (PRP)	CBT	PRP
Roberts 2010 [50]	The Aussie Optimism Program (AOP) program is based on PRP but targets anxiety as well as depression.	CBT	PRP
Rooney 2006 [51]	The Positive Thinking Program (PTP) program is based in part on the Aussie Optimism Program (AOP). It includes cognitive restructuring, and training in relaxation and distraction skills.	CBT	Positive Thinking Program
Sawyer 2010 [52]	The *beyondblue* schools research initiative utilised individual and ‘whole school’ focused components. The individual component aimed to improve problem solving and social skills, resilient thinking style and coping strategies. The whole school component included enhancements to the school climate to improve the quality of social interactions amongst all members of the school; improvements to care pathways to improve adolescents access to support and professional services; and community forums to provide adolescents, their families and school personnel to information about recognising problems and how to seek help.	CBT	beyondblue Schools Research Initiative
Seligman 1999 [53]	Intervention is based on CBT and similar PRP and includes cognitive restructuring, behavioural activation interventions including graded task breakdown, time management, anti-procrastination techniques, creative problem solving, assertiveness training, interpersonal skills including active listening, taking each other’s perspectives, controlling emotions, passive vs. assertive vs. aggressive behaviours, and relaxation training.	CBT	Unspecified
Seligman 2007 [54]	Replication of Seligman 1999 intervention with additional of web-based material and e-coaching primarily aimed at maintaining intervention effects over time.	CBT-partly online	Unspecified
Shatte 1997 [55]	Penn Resiliency Program (PRP)	CBT	PRP
Sheffield 2006 [56]	The Universal intervention included cognitive restructuring as well as problem solving interventions and was similar to the intervention described in Spence 2003. The indicated prevention program included these elements but also included interpersonal skills such as assertiveness, conflict resolution and negotiation and self-reward.	CBT	Problem Solving for Life
Spence 2003 [57]	The Problem Solving for Life (PSFL) program integrates cognitive restructuring and problem-solving skills training.	CBT	Problem Solving for Life
Stice a 2007 [58]	Based on the Coping with Stress program and focused on building rapport, increasing pleasant activities and cognitive restructuring.	CBT	Blues Program
Stice b 2007 [58]	Supportive-expressive group therapy, which aimed to establish and maintain rapport, provide support, and help the client identify and express emotions.	Other	
Stice c 2007 [58]	Bibliotherapy, which is the prescription of books for the treatment of a disorder.	Other	
Stice d 2007 [58]	Expressive writing in which participants write about issues of emotional significance to them.	Other	
Stice e 2007 [58]	Journalling	Other	
Stice a 2008 [59]	Based on the Coping with Stress program and focused on building rapport, increasing pleasant activities and cognitive restructuring	CBT	Blues Program
Stice b 2008 [59]	Supportive-expressive group therapy, which aims to establish and maintain rapport, provide support, and help the client identify and express emotions.	Other	
Stice c 2008 [59]	Cognitive Behavioural Bibliotherapy	Other	
Yu 2002 [60]	Chinese version of the Penn Resiliency Program (PRP)	CBT	PRP
Young 2006 [61]	The Interpersonal Therapy-Adolescent Skills Training (IPT–AST) program was created as an extension of interpersonal therapy. IPT–AST teaches communication and social skills necessary to develop and maintain positive relationships.	IPT	

ASQ: Attributional Style Questionnaire; CDI: Children’s depression Inventory; BDI: Beck Depression Inventory; CASQ: Childrens Attributional Style Questionnaire; CBCL-YSR: Child Behaviour Checklist-Youth Self Report; CES-D: Centre for Epidemiologic Studies Depression Scale; CIS-R: Clinical Interview Schedule-Revised; CPQ: child perception questionnaire (measures child’s perception of parental conflict); DSRS: Depression Self-Rating Scale RADS-2 : Reynolds Adolescent Depression Scale; RCADS: Revised Child Anxiety and Depression Scale; SBS-DES: Self-Report Questionnaire-Depression; SDIC: Short Depression Inventory for Children.

Sensitivity analysis allows investigation of the robustness of findings to decisions made about inclusion of studies. We had broad inclusion criteria and therefore we undertook sensitivity analyses using subgroup comparisons to assess the robustness of findings with regard to whether:

1. Interventions were delivered to universal *versus* targeted populations;
2. Interventions were delivered by a mental health clinician (including graduate level school counselors, school psychologists, cognitive and other therapists, clinical and other psychologists, psychiatric nurses, psychiatrist and mental health clinicians) *versus* students being trained in any of these mental health professions *versus* non mental health personnel;
3. Interventions included eight sessions or more *versus* less than eight sessions;
4. Outcomes were measured by the CDI/BDI *versus* the CES-D *versus* the RADS *versus* other measures.

### 2.6. Unit of Analysis Issues

In the references, letters after the year of publication indicate a separate study by the same author. In some cases a trial included multiple comparison arms that were relevant and in this case we have included these (dividing the control arm by the number of arms to which it was compared) and included a letter before the year of publication [36,58,59]. Finally, in some cases data were only provided for separate groups within a trial e.g., females and males, rather than totals so that in the analysis it is sometimes the case that the same reference appears to be repeated.

### 2.7. Heterogeneity

Heterogeneity was assessed on the basis of the Cochrane Handbook’s recommendations and I^2^ values are presented where I^2^ of 0–40%: might not be important; 30% to 60%: may represent moderate heterogeneity; 50% to 90%: may represent substantial heterogeneity; 75% to 100%: considerable heterogeneity. 

## 3. Results

### 3.1. Description of Studies

Complete details of the 43 trials included in this study are described in the Cochrane review [12]. Given the focus on exploring the effectiveness of different types of depression prevention programs, we have described the interventions tested in the trials included in this study below. 

Of the 50 intervention arms, 38 were classified as purely CBT; most were delivered to groups of adolescents in school settings, one was delivered online [26] and one was delivered partly online [53]. There was only one study that tested a purely IPT arm against a control [61]; one study included both a CBT treatment arm as well as an IPT treatment arm and a control group [39]; and two studies stated that their intervention incorporated CBT as well as IPT [20,48]. In this case we included the data from these studies in both the CBT and IPT subgroups (subgroups were not totaled). Eight intervention arms were classified as “other” (see Table 1). 

Of the named CBT programs, 12 trials reported in 11 papers tested the PRP [27,28,33,34,35,36,47,49,50,55,60], four tested the Coping with Stress program [30,31,32,39], three the FRIENDS program [21,42,43], two the LISA-T program [44,45], two the Problem Solving for Life program [56,57], two a stress inoculation approach [37,38], two the Blues program [58,59], one the Positive Thinking Program [51], one the Confident Kids program [22], one the Learn Young Learn Fair program [41], one the Teaching Kids to Cope [46], one the MoodGYM program [26], and one the RAP program [48], one was called the beyondblue schools research initiative [52], and a further six trials did not test a named program [20,23,24,29,40,54] (see Table 1). Overall, the content of these programs is largely similar; however, close inspection of the text describing each CBT intervention revealed some differences, albeit with some difficulties in accurately describing these given the non-standard descriptions and variable terminology. 

Every program included some form of cognitive restructuring. It wasn’t clear that all programs included typical behavioural activation; only 11 mentioned this specifically [22,23,26,29,32,48,54,56,59]. Specific mention of social skills training was made in a number of programs [23,38,54] and is part of the PRPs. Some of the unspecified programs used slightly different terminology such as interpersonal skills training [26,29,56] and assertiveness training [44,45], which was also stated as being included in one of the Coping with Stress programs [32]. Many stated that social or general problem solving was delivered in the program including the PRP, the Coping with Stress Program, MoodGYM [26], RAP [48], and the “Problem Solving for Life” program [56,57] and a number of “unspecified programs” [20,29,40,54]. None of the programs included any techniques or approaches used in 3rd wave CBT, such as mindfulness, acceptance, cognitive diffusion or distancing. 

Many CBT programs appear to include some focus on stress management including the PRPs, the Coping with Stress program, the Teaching Kids to Cope program [46], and the Positive Thinking Program [51]. Often these programs include relaxation as an intervention, as does MoodGYM [26], and RAP [48], and some unspecified programs [23,29,54]. Some of the programs are primarily anxiety management programs that were included in the review because they measure depression as an outcome. These include the FRIENDS program [21,42,43], the Confident Kids program [22], the Learn Young Learn Fair Program [41], and two studies by the author Hains [37,38].

Of the eight programs classified as ‘other’, one study had a ‘whole school’ program that aimed to change the school environment as well as delivering an intervention program to students [52]. One study provided a program that included some focus on parents and their parenting skills [34]. The two trials by Stice et al called The Blues Program had multiple treatment arms, including supportive-expressive group therapy, bibliotherapy, expressive writing, and journaling [58,59]. For further description of the trials and the interventions tested in these trials, see Table 2 and Characteristics of Included Studies in the full Cochrane review.

### 3.2. Assessment of Risk of Bias

Allocation concealment was unclear or not reported in the majority of studies and commonly participants and assessors were not blind to the treatment groups or blinding was unclear. A full description of the risk of bias in each study is given in the Characteristics of Included Studies in the full Cochrane review [12]. 

### 3.3. Effects of Intervention

#### 3.3.1. By *Type* of Intervention

##### CBT

There was evidence that the risk of having a depressive disorder was reduced by CBT post intervention, and at 3–9 month and 12 month follow-up. The level of depression symptoms was also reduced post intervention and at 12 month follow-up. However, there was a great deal of heterogeneity in findings (see Table 3).

**Table 2:** General characteristics of included studies.

Study Name	Size	Format	Targeted or Universal	Therapeutic Approach	Specific CBT Program Name	Number of Sessions	Manualised	Parent Component	Delivered by		Inclusion Criteria for Targeted Populations	Depression Outcome Measure
Arnarson 2009 [20]	171	Group	Targeted	CBT & IPT	Unspecified	14	Yes	No	Mental Health clinician		75th–90th percentile on CDI or >75th percentile on negative composite of the CASQ	CDI
Balle 2009 [21]	145	Group	Targeted	CBT	FRIENDS	6	Yes	No	Student mental health clinicians		High anxiety sensitivity	CDI
Berry 2009 [22]	54	Group	Targeted	CBT	Confident Kids	8	Yes	Yes	Student mental health clinicians		Anxiety symptoms	CES-D
Barnet 2007 [23]	84	Group	Targeted	Other		36	Yes	Yes	Non mental health personnel		Pregnant adolescents	CES-D
Bond 2004 [24]	2678	Group	Universal	Other		20	Yes	No	School teachers		NA	CIS-R
Cabiya 2008 [25]	278	Group	Targeted	CBT	Unspecified	12	Yes	No	Student mental health clinicians		Disruptive behaviour disorders	CDI
Calear 2009 [26]	1384	Individual	Universal	CBT-online	MoodGYM	5	Yes	No	Internet-based		NA	CES-D
Cardemil 2002 [27]	Trial 1: 49; Trial 2: 103	Group	Universal	CBT	PRP	12	Yes	No	Student mental health clinicians		NA	CDI
Chaplin 2006 [28]	234	Group	Universal	CBT	PRP	12	Yes	No	Both mental and non mental health personnel		NA	CDI
Clarke 1993 [29]	622	Group	Universal	CBT		3	Yes	No	Non mental health personnel		NA	CES-D
Clarke 1995 [30]	125	Group	Targeted	CBT	Coping with Stress	15	Yes	No	Mental health clinician		CES-D score of >=24	CES-D
Clarke 2001 [31]	94	Group	Targeted	CBT	Coping with Stress	15	Yes	Yes	Mental health clinician		CES-D score of ≥ 24 & parent with previous or current depressive episode	CES-D
Garber 2009 [32]	316	Group	Targeted	CBT	Coping with Stress	14	Unclear	Yes	Mental health clinician		CES-D score of ≥ 20 & parent with previous or current depressive episode	CES-D
Gillham 1995 [33]	143	Group	Targeted	CBT	PRP	12	Unclear	Yes. In the child-parent group only.	Student mental health clinicians		Children with summed z scores of ≤ 0.50 on CDI & CPQ	CDI
Gillham & Reivich 2006 [34]	44	Group	Targeted	CBT	PRP	8	Yes	Yes	Mental health clinician		High levels of depression and anxiety	CDI
Gillham & Hamilton 2006 [35]	271	Group	Targeted	CBT	PRP	12	Yes	No	Mental health clinician		CDI scores ≥ 7 for girls and ≥ 9 for boys	CDI
Gillham 2007 [36]	697	Group	Universal	CBT	PRP	12	Yes	No	Mental and non mental health personnel and students		NA	CDI
Hains 1990 [37]	24	Group	Universal	CBT	Stress Inoculation	5	Unclear	No	Mental health clinician		NA	BDI
Hains 1992 [38]	25	Group (plus 1 individual session)	Universal	CBT	Stress Inoculation	4	Unclear	No	Mental health clinician		NA	RADS
Horowitz a 2007 [39]	112	Group	Universal	CBT	Coping with Stress	8	Yes	No	Student mental health clinicians		NA	CES-D
Horowitz b 2007 [39]	99	Group	Universal	IPT	IPT-AST	8	Yes	No	Student mental health clinicians		NA	CES-D
Hyun 2005 [40]	32	Group	Targeted	CBT	Unspecified	8	Unclear	No	Mental health clinician	Runaway youth		BDI
Kraag 2009 [41]	1437	Group	Universal	CBT	Learn Young, Learn Fair	13	Yes	No	Non mental health personnel	NA		SDIC
Lock 2003 [42]	977	Group	Universal	CBT	FRIENDS	10	Yes	No	Student mental health clinicians	NA		CDI
Lowry-Webster 2001 [43]	594	Group	Universal	CBT	FRIENDS	10	Yes	Yes	Non mental health personnel	NA		CDI
Pössel 2004 [44]	342	Group	Universal	CBT	LISA-T	10	Yes	No	Mental health clinician	NA		CES-D
Pössel 2008 [45]	301	Group	Universal	CBT	LISA-T	10	Yes	No	Non mental health personnel with student mental health professionals	NA		SBB-DES
Puskar 2003 [46]	89	Group	Targeted	CBT	Teaching Kids to Cope	10	Unclear	No	Mental health clinician	RADS score ≥ 60		RADS
Quayle 2001 [47]	47	Group	Universal	CBT	PRP	8	Yes	No	Student mental health clinician	NA		CDI
Rivet-Duval 2010 [48]	160	Group	Universal	CBT & IPT	RAP	11	Yes	No	Non mental health personnel	NA		RADS
Roberts 2003 [49]	189	Group	Targeted	CBT	PRP	12	Yes	No	Mental health clinician	Elevated scores on the CDI		CDI
Roberts 2010 [50]	496	Group	Universal	CBT	Aussie Optimism Program	10	Yes	No	Non mental health personnel	NA		CDI
Rooney 2006 [51]	136	Group	Universal	CBT	Positive Thinking Program	8	Yes	No	Mental health clinician	NA		CDI
Sawyer 2010 [52]	5634	Group	Universal	CBT	beyondblue Schools Research Initiative	30	Yes	No	Non mental health personnel	NA		CES-D
Seligman 1999 [53]	231	Group	Targeted	CBT	Unspecified	8	Yes	No	Mental health clinician	Scored in the pessimistic quarter of the ASQ		BDI
Seligman 2007 [54]	227	Group	Targeted	CBT-partly online	Unspecified	8	Yes	No	Mental health clinician	BDI score of 9–24		BDI
Shatte 1997 [55]	152	Group	Universal	CBT	PRP	12	Yes	No	Non mental health personnel with student mental health professionals	NA		CDI
Sheffield 2006 [56]	2606	Group	Universal and targeted	CBT	Problem Solving for Life	8	Yes	No	Non mental health personnel for Universal; Mental health clinician for targeted	Score in the top 20% on the combined scores on the CDI & CES-D.		CDI
Spence 2003 [57]	1234	Group	Universal	CBT	Problem Solving for Life	8	Yes	No	Non mental health personnel	NA		BDI
Stice a 2007 [58]	50	Group (CBT)	Targeted	CBT	Blues Program	4	Yes	No	Student mental health clinician	CES-D score of ≥ 20		BDI
Stice b 2007 [58]	19	Group (Supportive expressive)	Targeted	Other		4	Yes	No	Student mental health clinician	CES-D score of ≥ 20		BDI
Stice c 2007 [58]	28	Individual (Bibliotherapy)	Targeted	Other		Not specified	Yes	No	Self-led	CES-D score of ≥ 20		BDI
Stice d 2007 [58]	27	Individual (Expressive writing)	Targeted	Other		4	Yes	No	Self-led	CES-D score of ≥ 20		BDI
Stice e 2007 [58]	34	Individual (Journaling)	Targeted	Other		Not specified	Yes	No	Self-led	CES-D score of ≥ 20		BDI
Stice a 2008 [59]	89	Group (CBT)	Targeted	CBT	Blues Program	6	Yes	No	Student mental health clinician	CES-D score of ≥ 20		CES-D
Stice b 2008 [59]	88	Group (Supportive Expressive)	Targeted	Other		6	Yes	No	Student mental health clinician	CES-D score of ≥ 20		CES-D
Stice c 2008 [59]	80	Individual (Bibliotherapy)	Targeted	Other		Not specified	Yes	No	Self-led	CES-D score of ≥ 20		CES-D
Yu 2002 [60]	270	Group	Targeted	CBT	PRP	10	Yes	No	Non mental health personnel	Elevated scores on the CDI and the Cohesion and Conflict subscales of the Family Environment scale		CDI
Young 2006 [61]	41	Group	Targeted	IPT		10	Yes	No	Mental health clinician	CES-D score of 16–39		CES-D

ASQ: Attributional Style Questionnaire; CDI: Children’s depression Inventory; BDI: Beck Depression Inventory; CASQ: Childrens Attributional Style Questionnaire; CBCL-YSR: Child Behaviour Checklist-Youth Self Report; CES-D: Centre for Epidemiologic Studies Depression Scale; CIS-R: Clinical Interview Schedule-Revised; CPQ: child perception questionnaire (measures child’s perception of parental conflict); DSRS: Depression Self-Rating Scale RADS-2: Reynolds Adolescent Depression Scale; RCADS: Revised Child Anxiety and Depression Scale; SBS-DES: Self-Report Questionnaire-Depression; SDIC: Short Depression Inventory for Children.

##### IPT

There were two studies that included an intervention that contained elements of both CBT and IPT interventions [20,48], one study that included one arm that tested a purely IPT intervention [39] and one study that tested the efficacy of IPT with a control group [61]. Two studies that included an intervention which contained elements of both CBT and IPT showed that there was no evidence that interventions that included IPT reduced the risk of having a depressive disorder post intervention; however, at 3–9 months there was evidence that interventions that included IPT reduced the risk of depressive disorder, with no heterogeneity evident at this time point. The Young 2006 study [61], which was purely IPT, was combined with the Rivet-Duval 2010 study [48] (which also included elements of CBT) and there was evidence that interventions that include elements of IPT reduced depression symptoms at post intervention, although there was significant heterogeneity and the evidence of effect did not remain at 3–9 month follow-up. Only the Young 2006 study [61] measured depression symptoms at 12-month follow-up and there was no evidence of effect (see Table 3). 

##### Other

There was little evidence that these programs reduced the risk of depressive disorder or reduced depressive symptoms compared with no intervention post intervention, at 3–9 month or 12 month follow-up. 

##### Test for Differences between Types of Intervention

There was evidence that the treatment effect for depressive disorder was modified by the type of therapeutic approach taken to depression prevention at post intervention and 12 month follow-up, and for depression symptoms at 12-month follow-up (see Table 3). When those studies that included both CBT and IPT interventions were excluded in sensitivity analysis, these subgroup differences remained.

**Table 3:** Summary of meta-analysis results for the therapeutic approach to prevention programs.

Program	Post Intervention		3–9 Month Follow-up		12-Month Follow-up	
	Depressive disorder (RD)	Depression symptoms (SMD)	Depressive disorder (RD)	Depression symptoms (SMD)	Depressive disorder (RD)	Depression symptoms (SMD)
CBT	14 studies; 16 intervention arms; N = 1776 **RD −0.11; 95% CI −0.17 to −0.05** I^2^ = 66%	39 studies; 39 intervention arms; N = 11630 **SMD −0.12; 95% CI −0.24 to −0.01** I^2^ = 86%	14 studies; 18 intervention arms; N = 2254 **RD −0.11; 95% CI −0.15 to −0.06** I^2^ = 46%	27 studies; 33 intervention arms; N = 6351 SMD −0.09; 95% CI −0.25 to 0.07 I^2^ = 87%	9 studies; 10 intervention arms; N = 1149 **RD−0.08; 95% CI −0.16 to -0.00** I^2^ = 75%	16 studies; 21 intervention arms; N = 5047 **SMD −0.11; 95% CI −0.17 to −0.04** I^2^ = 13%
IPT	2 studies; 2 intervention arms; N = 265 RD 0.09; 95% CI −0.35 to 0.17	3 studies; 3 intervention arms; N = 327**SMD −0.54; 95% CI −0.94 to −0.13** I^2^ = 67%	2 studies; 2 intervention arms; N = 252 **RD −011; 95% CI −0.19 to −0.04** I^2^ = 0%	3 studies; 4 intervention arms; N = 327 SMD −0.26 [−0.62, 0.10]	NA	1 study; 1 intervention; N = 41 SMD −0.56 [−1.22, 0.10]
Other	4 studies; 5 intervention arms; N = 1843 RD −0.01; 95% CI −0.05 to 0.02	5 studies; 9 intervention arms; N = 2178 **SMD −0.21; 95% CI −0.39 to −0.03** I^2^ = 52%	3 studies; 5 intervention arms; N = 623 RD −0.02; 95% CI −0.11 to 0.07	4 studies; 9 intervention arms; N = 766 SMD −0.08; 95% CI −0.23 to 0.07	2 studies; 2 intervention arms; N = 1363 RD 0.01; 95% CI −0.03, 0.05	2 studies; 2 intervention arms; N = 1375 **SMD 0.14; 95% CI 0.03 to 0.24** I^2^ =0%
Subgroup differences	**χ^2^ = 8.86,**	χ^2^ = 2.85,	χ^2^ = 3.18,	χ^2^ = 0.83,	**χ^2^ = 4.13,**	**χ^2^ = 17.07,**
	***p* = 0.01**	*p* = 0.24	*p* = 0.20	*p* = 0.66	***p* = 0.04**	***p* = 0.0002**

RD: Risk Difference; SMD: Standardised Mean Difference; Bold font indicates significant results.

#### 3.3.2. By CBT Program Type

##### Penn Resiliency Program (PRP)

This program is the most studied intervention of all the named CBT programs with 10 studies of this program or its derivatives. Overall, the risk of depressive disorder was reduced at all three time points and depressive symptoms were also reduced at all three time points (see Table 4). There is considerable heterogeneity associated with the results for reduction in depressive disorder at post intervention and 3 to 9 months follow-up, which indicates considerable variation across studies. For example, results based on data from the African American sample in the study by Cardemil [27] are quite different to that of the Latino study to study; and the results for the child only intervention group are different to that of the parent and child intervention group in study by Gillham 1995 [33]. However, even after removing these data, there remains considerable unexplained statistical heterogeneity. 

##### Coping with Stress

Four studies tested the effectiveness of this intervention. There was evidence that it reduced the risk of having a depressive disorder post intervention and at longer-term follow-up. There was also evidence that this program reduced depression symptoms post intervention but not at 3 to 9 or 12 month follow-up. Heterogeneity was high for depressive disorder post intervention (see Table 4). 

**Table 4:** Summary of meta-analysis results for named programs.

Program	Post Intervention		3–9 Month Follow-up		12-Month Follow-up	
	Depressive disorder (RD)	Depression symptoms (SMD)	Depressive disorder (RD)	Depression symptoms (SMD)	Depressive disorder (RD)	Depression symptoms (SMD)
PRP	6 studies; 8 intervention arms; N = 483 **RD −0.18** **[−0.31, −0.05]** I^2^ = 74%	11 studies; 12 intervention arms; N = 1628 **SMD 0.11** **[−0.21, 0.00]** I^2^ = 0%	5 studies; 6 intervention arms; N = 363 **RD −0.19** **[−0.36, −0.01]** I^2^ = 84%	10 studies; 13 intervention arms; N = 1206 **SMD −0.17** **[−0.29, −0.05]** I^2^ = 0%	4 studies; 5 intervention arms; N = 273 **RD −0.05** **[−0.14, −0.03]** I^2^ = 16%	7 studies; 10 intervention arms; N = 926 **SMD −0.18 [−0.31, -0.05]** I^2^ = 0%
Coping with Stress	2 studies; 2 intervention arms; N = 215 **RD −0.16 [−0.27, −0.04]** I^2^ = 49%	4 studies; 4 intervention arms; N = 598 **SMD −0.34** **[−0.50, −0.17]** I^2^ = 0%	2 studies; 2 intervention arms; N = 427 **RD −0.12 [−0.19, −0.04]** I^2^ = 0%	3 studies; 3 intervention arms; N = 494 SMD −0.14 [−0.32, 0.04]	2 studies; 2 intervention arms; N = 195 **RD −0.12 [−0.24, −0.01]** I^2^ = 0%	2 studies; 2 intervention arms; N = 196 SMD −0.25 [−0.77, 0.27]
Friends	1 study; 1 intervention arm; N = 239 RD −0.06 [−0.17, 0.04]	3 studies; 3 intervention arms; N = 486 SMD −0.09 [−0.28, 0.09]	NA	1 study; 1 intervention arm; N = 68 SMD 0.19 [−0.29, 0.67]	2 studies; 2 intervention arms; N = 452 RD −0.12 [−0.57, 0.33]	2 studies; 2 intervention arms; N = 418 **SMD −0.27 [−0.47, −0.06]** I^2^ = 0%
Positive Thinking Program	1 study; 1 intervention arm; N = 76 RD −0.10 [−0.25, 0.05]	1 study; 1 intervention arm; N = 76 **SMD −0.57 [−1.04, −0.10]**	1 study; 1 intervention arm; N = 75 **RD −0.21 [−0.37, −0.05]**	1 study; 1 intervention arm; N = 75 SMD −0.25 [−0.71, 0.21]	NA	NA
Blues Program	NA	2 studies; 2 intervention arms; N = 153 **SMD −0.65** **[−1.03, −0.26]** I^2^ = 0%	1 study; 1 intervention arm; N = 100 RD −0.09 [−0.25, 0.07]	2 studies; 2 intervention arms; N = 153 **SMD −0.38** **[−0.76, −0.00]** I^2^ = 0%	NA	
Aussie Optimism Program	NA	1 study; 1 intervention arm; N = 427 SMD 0.14 [−0.05, 0.33]	NA	1 study; 1 intervention arm; N = 395 SMD 0.12 [−0.08, 0.32]	NA	
Stress focus	NA	2 studies; 2 intervention arms; N = 38 SMD −0.47 [−1.17, 0.23]	NA	NA	NA	NA
Confident kids	NA	1 study; 1 intervention arm; N = 44 SMD −0.66 [−1.58, 0.25]	NA	NA	NA	NA
Learn Young Learn Fair	NA	1 study; 1 intervention arm; N = 1102 SMD 0.00 [−0.12, 0.12]	NA	NA	NA	1 study; 1 intervention arm; N = 1011 SMD −0.02 [−0.15, 0.10]
Teaching Kids to Cope	NA	1 study; 1 intervention arm; N = 80 **SMD −0.47 [−0.92, −0.03]**	NA	1 study; 1 intervention arm; N = 76 **SMD −0.49** **[−0.95, −0.04]**	NA	1 study; 1 intervention arm; N = 70 SMD −0.30 [−0.77, 0.17]
Moodgym	NA	1 study; 1 intervention arm; N = 719 SMD −0.15 [−0.30, 0.00]	NA	1 study; 1 intervention arm; N = 690 SMD −0.13 [−0.28, 0.03]	NA	NA
LISA-T	NA	2 studies; 2 intervention arms; N = 446 SMD −0.07 [−0.26, 0.11]	NA	2 studies; 2 intervention arms; N = 435 SMD −0.23 [−0.65, 0.20]	NA	NA
Problem solving for Life	NA	2 studies; 4 intervention arms; N = 2310 **SMD −0.14 [−0.25, −0.04]** I^2^ = 29%	1 study; 3 intervention arms; N = 714 RD −0.06 [−0.12, 0.01]	1 study; 3 intervention arm; N = 1843 SMD −0.03 [−0.14, 0.08]	1 study; 1 intervention arm; N = 229 RD 0.01 [−0.06, 0.09]	2 studies; 4 intervention arms; N = 2207 SMD 0.00 [−0.09, 0.10]
RAP	1 study; 1 intervention arm; N = 116 **RD −0.17 [−0.33, −0.01]**	1 study; 1 intervention arm; N = 116 SMD −0.32 [−0.68, 0.05]	1 study; 1 intervention arm; N = 116 RD −0.10 [−0.28, 0.07]	1 study; 1 intervention arm; N = 116 SMD −0.03 [−0.39, 0.34]	NA	NA
Unspecified	3 studies; 4 intervention arm; N = 667 RD −0.03 [−0.06, 0.01]	5 studies; 7 intervention arms; N = 956 **SMD −0.26 [−0.50, −0.02]** I^2^ = 63%	2 studies; 3 intervention arms; N = 409 **RD −0.09 [−0.15, −0.03]** I^2^ = 0%	3 studies; 4 intervention arms; N = 717 SMD −0.24 [−0.54, −0.05]	NA	1 study; 1 intervention arm; N = 219 SMD −0.25 [−0.52, 0.02]
Subgroup differences	χ^2^ = 10.64,	**χ^2^ = 36.31,**	χ^2^ = 4.96,	χ^2^ = 16.18,	χ^2^ = 4.47,	**χ^2^ = 13.05,**
	*p* = 0.06	***p* = 0.0009**	*p* = 0.55	*p* = 0.13	*p* = 0.21	***p* = 0.04**

RD: Risk Difference; SMD: Standardised Mean Difference; Bold font indicates significant results.

##### Friends

Three studies investigated the effectiveness of this intervention. There was no evidence that it reduced the risk of depressive disorder at any time point. Likewise, three studies reporting on depressive symptoms post intervention, and one study reporting on this outcome at 3–9 months follow-up showed no evidence that this program reduced depressive symptoms. At 12-month follow-up 2 studies showed some evidence of a reduction in depressive symptoms (see Table 4).

##### Positive Thinking Program

One study of this intervention showed there was no evidence that this program reduced depressive disorder at post intervention; however, by 3–9 months follow-up there was some evidence of effect. There was evidence from this same study that the program reduced depression symptoms at post intervention; however, the effect was no longer evident at follow-up (see Table 4). 

##### Blues Program

Two studies of this intervention showed there was some evidence that this program reduced depression symptoms post intervention and at 3–9 months follow-up with no evidence of heterogeneity. There was no evidence that it reduced depressive disorder at any time point (see Table 4).

##### Aussie Optimism Program

One study of this program showed no evidence of this program in reducing depression symptoms or disorder at any time point (see Table 4). 

##### Stress Inoculation (General)

Two very small studies by the same author tested the effectiveness of a program based on the concept of stress inoculation but did not measure depressive disorder. There was no evidence that this program reduced depression symptoms post intervention with no follow-up measurement (see Table 4). 

##### Confident Kids

This program was tested in one small study that measured depression symptoms post intervention and showed no evidence of effect (see Table 4). 

##### Learn Young, Learn Fair

One large study tested this intervention and showed no evidence of effect on reducing depression symptoms post intervention or at 12-month follow-up (see Table 4). 

##### Teaching Kids to Cope

One study tested this program and while not measuring depressive disorder, did show that this program was effective in reducing depression symptoms post intervention and at 3–9 months follow-up but not at 12-month follow-up (see Table 4). 

##### MOOD-GYM

One large study investigating this program showed there was no evidence of the program’s effectiveness in reducing depression symptoms at post intervention or at 3–9 months follow-up (see Table 4). 

##### LISA-T

There were two studies that tested the effectiveness of this program in reducing depression symptoms that showed no evidence of its effectiveness post intervention or at 3–9 months follow-up and no 12-month follow-up data (see Table 4). 

##### Problem Solving for Life

There was some evidence of the effect of this program in reducing the risk of depressive disorder at 3–9 month that did not reach significance but this effect was not evident at 12-months. While two studies did show some evidence of the effectiveness of this program in reducing depression symptoms post intervention, the effect was no longer evident at 12 month follow-up (see Table 4). 

##### RAP

One study testing the effectiveness of this program showed evidence of a reduction in the risk of depressive disorder post intervention; however, this effect was no longer evident at 3–9 months follow-up (there was no 12-month follow-up data). There was no evidence of the effectiveness of this program in reducing depression symptoms at any time point (see Table 4). 

##### Unspecified

While there was no evidence that this group of general CBT programs reduced the risk of depressive disorder post intervention; there was some evidence of effect of this group of programs at 3–9 month follow-up with no evidence of heterogeneity. No study measured this outcome at 12-month follow-up. There was some evidence of the effectiveness of these general CBT programs in reducing depression symptoms post intervention with evidence of heterogeneity. This effect was not evident at follow-up (see Table 4).

##### Test for Differences Between Types of Named Programs

There was evidence that the effect on depression symptoms post intervention and at 12 months follow-up was modified by the type of CBT program but not at 3 to 9 months follow-up or for depressive disorder (see Table 4). 

### 3.4. Sensitivity Analyses

The planned sensitivity analyses showed that very few factors modified overall treatment effects. Results indicated a small but significant positive effect of universal interventions in reducing depressive disorder but not depression symptoms post intervention and at 3 to 9 month follow-up and in reducing symptoms but not depressive disorder at 12-month follow-up. There were small but significant positive effects of targeted interventions in reducing both depressive disorder and symptoms at all time points. Even though there were no significant differences between the universal intervention and comparison group at some time points, the direction of treatment effect favoured the universal intervention. The subgroup analysis showed that the overall treatment effects (for universal and targeted interventions combined) were not modified by delivery to a universal or targeted population (See Table 5); that is to say, there is no significant variation in mean effects in the different subgroups. The delivery of 8 or more sessions was more effective in reducing depressive disorder but not depression symptoms at all time points, whereas fewer than 8 sessions had no impact on outcomes; however, the overall treatment effects were not modified by the number of sessions delivered (See Table 6). Delivery of programs by mental health professions reduced depressive disorder and symptoms at all time points, whereas delivery by a non mental health expert only reduced depressive disorder post intervention and at 3 to 9 months follow-up and delivery by a student only resulted in reduced depressive disorder post intervention and depressive symptoms at post intervention and 3 to 9 months follow-up. Again, however, there was no evidence that who delivered the intervention modified the treatment effect (See Table 7). There was evidence that the type of outcome measurement used to measure depression symptoms modified the findings post intervention but not at other time points. When the CDI or BDI was used (these were the most commonly used tools) depressive symptoms were shown to be reduced at 12 months only; when the CES-D was used, symptoms were reduced post intervention and at 3 to 9 months follow-up but not at 12 months; when the RADS was used symptoms were reduced only at post intervention, however, the largest effect size was found on the RADS at this time point (see Table 8). Use of other tools did not show reductions in depressive symptoms at any time point.

**Table 5:** Summary of meta-analysis results for approach to prevention programs analysed by population.

Program	Post Intervention		3–9 Month Follow-up		12-Month Follow-up	
	Depressive disorder (RD)	Depression symptoms (SMD)	Depressive disorder (RD)	Depression symptoms (SMD)	Depressive disorder (RD)	Depression symptoms (SMD)
Universal	8 studies; 9 intervention arm; N = 1025 **RD −0.14 [−0.23, −0.06]** I^2^ = 68%	21 studies; 26 intervention arm; N = 6519 SMD −0.05 [−0.23, 0.13]	8 studies; 10 intervention arm; N = 1228 **RD −0.13 [−0.21, −0.06]** I^2^ = 70%	14 studies; 18 intervention arm; N = 4077 SMD −0.02 [−0.27, 0.23]	6 studies; 7 intervention arm; N = 910 RD −0.06 [−0.15, 0.03]	10 studies; 14 intervention arm; N = 3737 **SMD −0.08 [−0.14, −0.01]** I^2^ = 68%
Targeted	6 studies; 7 intervention arms; N = 751 **RD −0.09 [−0.16, −0.01]** I^2^ = 53%	18 studies; 21 intervention arms; N = 3363 **SMD −0.25 [−0.37, −0.14]** I^2^ = 44%	7 studies; 9 intervention arms; N = 1255 **RD −0.09 [−0.14, −0.05]** I^2^ = 0%	13 studies; 16 intervention arms; N = 2880 **SMD −0.18 [−0.30, −0.07]** I^2^ = 41%	3 studies; 3 intervention arm; N = 239 **RD −0.14 [−0.24, −0.04]** I^2^ = 0%	7 studies; 8 intervention arm; N = 1902 **SMD −0.14 [−0.28, 0.00]** I^2^ = 41%
Subgroup differences	χ^2^ = 0.99,	χ^2^ = 3.37,	χ^2^ = 0.75,	χ^2^ = 1.39,	χ^2^ = 1.38,	χ^2^ = 0.61,
	*p* = 0.32	*p* = 0.07	*p* = 0.39	*p* = 0.24	*p* = 0.24	*p* = 0.44

RD: Risk Difference; SMD: Standardised Mean Difference; Bold font indicates significant results.

**Table 6:** Summary of meta-analysis results for approach to prevention programs analysed by number of sessions delivered.

Sessions	Post Intervention		3–9 Month Follow-up		12-Month Follow-up	
	Depressive disorder (RD)	Depression symptoms (SMD)	Depressive disorder (RD)	Depression symptoms (SMD)	Depressive disorder (RD)	Depression symptoms (SMD)
8 or more	13 studies; 15 intervention arm; N = 1503 **RD −0.13 [−0.20, −0.07]** I^2^ = 71%	32 studies; 39 intervention arm; N = 8014 SMD −0.13 [−0.28, 0.01]	12 studies; 16 intervention arm; N = 1881 **RD −0.12 [−0.17, −0.07]** I^2^ = 55%	22 studies; 28 intervention arm; N = 5167 SMD −0.09 [−0.28, 0.10]	9 studies; 10 intervention arms; N = 1149 **RD −0.08 [−0.16, −0.00]** I^2^= 75%	16 studies; 21 intervention arm; N = 5047 SMD −0.11 [−0.17, −0.04]
<8	1 study; 1 intervention arm; N = 273 RD −0.01 [−0.11, 0.09]	7 studies; 7 intervention arms; N = 1251 SMD −0.17 [−0.36, 0.02]	2 studies; 2 intervention arms; N = 373 RD −0.07 [−0.14, 0.01]	5 studies; 5 intervention arms; N = 1184 SMD −0.13 [−0.26, 0.00]	0 studies	0 studies
	Depressive disorder (RD)	Depression symptoms (SMD)	Depressive disorder (RD)	Depression symptoms (SMD)	Depressive disorder (RD)	Depression symptoms (SMD)
Subgroup differences	**χ^2^ = 4.50, ** ***p* = 0.03**	χ^2^ = 0.09, *p* = 0.76	χ^2^ = 1.28, *p* = 0.26	χ^2^ = 0.09, *p* = 0.76	NA	NA

RD: Risk Difference; SMD: Standardised Mean Difference; Bold font indicates significant results.

**Table 7:** Summary of meta-analysis results for approach to prevention programs analysed by who delivered the intervention.

Delivery	Post Intervention		3–9 Month Follow-up		12-Month Follow-up	
	Depressive disorder (RD)	Depression symptoms (SMD)	Depressive disorder (RD)	Depression symptoms (SMD)	Depressive disorder (RD)	Depression symptoms (SMD)
Mental health expert	5 studies; 5 intervention arm; N = 665 **RD −0.10 [−0.18, −0.01]** I^2^ = 71%	15 studies; 15 intervention arm; N = 2649 **SMD −0.24 [−0.37, −0.11]** I^2^ = 54%	5 studies; 5 intervention arm; N = 883 **RD −0.11 [−0.16, −0.06]** I^2^ = 0%	12 studies; 12 intervention arm; N = 2612**SMD −0.21 [−0.33, −0.09]** I^2^ = 52%	2 studies; 2 intervention arm; N = 195 **RD −0.12 [−0.24, −0.01]** I^2^ = 0%	8 studies; 8 intervention arm; N = 1718**SMD −0.17 [−0.29, −0.05]** I^2^ = 21%
Non mental health expert	4 studies; 4 intervention arms; N = 597 **RD −0.18 [−0.35, −0.02]** I^2^ = 80%	14 studies; 15 intervention arms; N = 5267 SMD 0.04 [−0.20, 0.28]	5 studies; 5 intervention arms; N = 1065 **RD -0.13 [−0.22, −0.05]** I^2^ = 63%	8 studies; 8 intervention arms; N = 2861 SMD 0.12 [−0.24, 0.47]	4 studies; 4 intervention arm; N = 595 RD −0.02 [−0.09, 0.04]	7 studies; 8 intervention arm; N = 3397 SMD −0.06 [−0.13, 0.01]
Student	5 studies; 5 intervention arms; N = 514 **RD −0.08 [−0.13, −0.03]** I^2^ = 0%	12 studies; 13 interventionarms; N = 1143 **SMD −0.24** **[−0.41, −0.07]** I^2^ = 36%	5 studies; 5 intervention arms; N = 306 RD −0.08 [−0.18, 0.02]	8 studies; 9 intervention arms; N = 597 **SMD −0.19 [−0.37, −0.00]** I^2^ = 15%	3 studies; 3 intervention arm; N = 359 RD −0.09 [−0.28, 0.11]	3 studies; 3 intervention arms; N = 340 SMD −0.21 [−0.53, 0.10]
Subgroup differences	χ^2^ = 1.30, *p* = 0.52	χ^2^ = 4.44, *p* = 0.11	χ^2^ = 0.55, *p* = 0.76	χ^2^ = 2.82, *p* = 0.24	χ^2^ = 2.55, *p* = 0.28	χ^2^ = 2.79, *p* = 0.25

RD: Risk Difference; SMD: Standardised Mean Difference; Bold font indicates significant results.

Further sensitivity analyses were undertaken where the effect of moderators was explored separately in programs delivered to universal populations and for programs delivered to targeted populations. This was undertaken on the basis that the factors that may modify overall treatment effect systematically differed across universal and targeted programs, for example, targeted programs were most often delivered by mental health clinicians, whereas, universal programs were mostly delivered by non mental health clinicians and students (See Table 2). 

The overall effect of targeted programs on depression symptoms was modified by the type of therapeutic approach taken to depression prevention (CBT *vs*. IPT *vs*. other) post intervention and at 3 to 9 month but the treatment effect on depressive disorder was not modified by the therapeutic approach. For universal programs, the overall effect on depressive disorder was modified by the therapeutic approach post intervention and at 3 to 9 months but not at 12 months and the overall effect on symptoms was modified at 12 months only.

**Table 8:** Summary of meta-analysis results for approach to prevention programs analysed by the outcome measurement tool used.

Tool	Post Intervention	3–9 Month Follow-up	12-Month Follow-up
	Depression Symptoms (SMD)	Depression Symptoms (SMD)	Depression Symptoms (SMD)
CDI/BDI	24 studies; 30 intervention arm; N = 5686 SMD −0.06 [−0.26, 0.13] I^2^ = 90%	17 studies; 22 intervention arm; N = 4085 SMD −0.05 [−0.29, 0.19]I^2^ = 91%	12 studies; 17 intervention arm; N = 3770 **SMD −0.10 [−0.18, -0.03]** I^2^ = 4%
CES-D	9 studies; 10 intervention arms; N = 2022 **SMD −0.24 [−0.35, −0.13]** I^2^ = 17%	7 studies; 8 intervention arms; N = 1832 **SMD −0.18 [−0.29, −0.06]** I^2^ = 23%	2 studies; 2 intervention arm; N = 196 SMD −0.25 [−0.77, 0.27]
RADS	3 studies; 3 intervention arms; N = 213**SMD −0.42 [−0.69, −0.14]**I^2^ = 0%	2 studies; 2 intervention arms; N = 192SMD −0.24 [−0.69, 0.22]	1 study; 1 intervention arms; N = 70 SMD −0.30 [−0.77, 0.17]
Other	3 studies; 3 intervention arms; N = 1344 SMD −0.00 [−0.11, 0.11]	1 study; 1 intervention arm; N = 242 SMD −0.02 [−0.27, 0.24]	1 study; 1 intervention arm; N = 1011 SMD −0.02 [−0.15, 0.10]
Subgroup differences	**χ^2^ = 14.45, *p* = 0.002**	χ^2^ = 2.11, *p* = 0.55	χ^2^ = 2.46, *p* = 0.48

RD: Risk Difference; SMD: Standardised Mean Difference; Bold font indicates significant results.

For targeted programs, the overall effect on depression symptoms was modified by the specific program type only at post intervention (and no longer at 12 months); and for universal programs there was no evidence that the specific type of program modified the overall treatment effect. The numbers of studies in each subgroup was greatly reduced by splitting the analysis. 

While we found no modifying effect on outcome of the number of sessions delivered when universal and targeted programs were analysed together; when investigated separately, the overall effect on depression symptoms of targeted programs was modified by the number of sessions delivered post intervention only. For universal programs, the overall treatment effect on depressive disorder was modified by the number of sessions delivered post intervention only. In both cases, delivery of 8 or more sessions resulted in a significantly greater reduction in symptoms or depressive disorder respectively, whereas there was no difference in outcomes when fewer than 8 sessions were delivered. Again the small number of studies, particularly in the subgroup in which the intervention comprised fewer than 8 sessions should be noted; indeed often there were no data available for these intervention. 

There was no evidence the treatment effect was modified by who delivered the intervention for either targeted or for universal programs. Similarly, there was no evidence that the tool that was used to measure outcome modified the treatment effect when targeted and universal programs were considered separately, in contrast to when they were considered together. 

## 4. Discussion

### 4.1. Principal Findings

While overall the findings indicate small but significant effect sizes suggesting a small but positive effect on reducing depression symptoms and disorders, one of the most striking findings from this exploratory re-analysis of depression prevention programs is the variation in outcome across trials. There is some evidence that more consideration should be given to the specific therapeutic approach used in depression preventions programs. CBT is the most studied type of intervention and there is some evidence of its efficacy in reducing the risk of developing a depressive disorder and reducing depression symptoms. IPT appears promising from the trials that included intervention arms using a purely IPT based intervention [39,61]; two combined IPT with CBT but it is impossible to tease out the differential effects of the IPT approach from these trials. A number of other trials that tested a range of interventions showed little consistent evidence of effectiveness. 

While this review shows some evidence of the efficacy of CBT based depression prevention interventions, there was significant statistical heterogeneity across these trials (of CBT) at most time points and we sought to explore this by investigating whether each of the different CBT programs modified the overall effect of CBT. Overall the treatment effect for depression symptoms (but not disorder) was modified by the specific type of program used; however, when targeted and universal programs were considered separately, this effect was less apparent only being seen for targeted programs at the post intervention time point. This may be because there were few universal programs that used the Coping with Stress Course. Overall, there was limited power for these analyses when potential modifiers were considered separately for targeted and universal programs because the majority of programs have been tested in only a handful of trials. 

Nevertheless, as far as targeted programs are concerned for individual results, The Penn Resiliency Program is the most studied of the named CBT prevention programs and showed consistent results across outcomes and time points, albeit with heterogeneity demonstrated for the outcome of depressive disorder across, particularly for the universal studies, (compared to Coping with Stress). Our results are consistent with another review of the PRP [15]. This program has a considerable focus on cognitive skills, as well as including a focus on problem solving. 

The Coping with Stress program also has impressive results in terms of preventing the onset of depressive disorder up to 12 months follow-up, as well as reducing depression symptoms at post intervention and 3–9 months follow-up. This program focuses almost entirely on cognitive restructuring skills, with only three trials of this program including behavioural activation [22,59], one of which was only in the continuation phase [32]. However, one of the issues with this program is that not only has it mostly been delivered as a targeted intervention, but in several of the studies of Coping with Stress participants were included after a two-stage screening procedure to identify adolescents with at least one parent with a history of depression as well as elevated depression symptoms. In one study [32] this resulted in the identification of a large number of young people who had previously suffered from depressive disorder, so that this “prevention” program was strictly a relapse prevention program rather than primary prevention. We are also aware that a recent study that we were unable to include in this analysis tested this intervention in a real world effectiveness study in Santiago, Chile and there was no significant effect of the program in preventing depression [62].

There were too few studies of the remaining CBT programs and the results varied across outcomes and time points so that robust conclusions about their effectiveness cannot be drawn. It is worth noting three of these programs as having some initial evidence of effectiveness. The Blues Program includes only four sessions and focuses on cognitive restructuring and includes behavioural activation and results indicated a small and significant effect in reducing depression symptoms post intervention and at 3 to 9 months follow-up with no effect of depressive disorder. The Teaching Kids to Cope Program emphasizes skills to cope with stress, including social skills training, assertiveness training, conflict resolution and relaxation. However, the incidence of depressive disorder was not measured. The Positive Thinking Program is based on the “Aussie Optimism Program” [50], which included cognitive restructuring, and training in relaxation and distraction skills. While the results indicated a small and significant effect reducing the risk of the onset of depressive disorder at 3–9 months follow-up, it was small study.

There was some evidence for the effectiveness of the group of programs we classified as “unspecified” at post intervention and 3 to 9 month follow-up. One mentioned the Coping with Stress program [20], but also included elements of interpersonal therapy and problem solving. Three included a major [40] or some aspects of social skills training [29,54]. The study reported in Clarke included a psychoeducation intervention [29]. Hyun [40] was aimed at preventing depression in runaway youth and included cognitive restructuring, behavioural activation and relaxation with a focus on aspects of homelessness.

While some reviews have suggested that targeted but not universal prevention programs are effective [7,8], and the results from this study at individual time points more often reveal significant findings for the targeted programs; the current study showed that the results were not moderated by type of prevention (universal *versus* targeted). It is important to realize that targeted interventions are more likely to show effect simply because they start with a group of young people with higher baseline levels of depression. The fact that there is so little difference between findings for universal and targeted approaches should lead us to be cautious in assuming superiority of one approach over the other, particularly as targeted interventions are often harder to implement, and risk missing a large number of people who could benefit from the intervention [63].

The measurement tool that is used may have an impact on findings, with the RADS being associated with the largest effect sizes when all the studies are considered together. This may be because of the psychometric properties in community populations. The change in scores on the RADS is generally small compared with the standard deviation whereas the change scores on the CDI and BDI are large compared with the standard deviation (for example see Merry *et al*. [64]). RADS is recommended as a good measure of depression in community populations [65]. The studies of the Coping with Stress programs all used CES-D as an outcome measurement, although the most robust findings are derived from a measure of period prevalence of depressive disorder. The PRP programs used the CDI or BDI. The CDI, which was designed for a clinical rather than a community population, has been criticized for its less than optimal ability to detect treatment effects, particularly in non-clinical populations [65]. Interestingly, when investigated separately for targeted and universal intervention studies, the type of tool used was no longer a significant modifier of treatment effects. 

When analysed separately, the number of sessions delivered did modify the treatment effects post intervention for both targeted and universal interventions. This finding is consistent with a meta-analysis of 69 programs to reduce depression in children, adolescents, adults and older adults. In this analysis interventions of more than eight sessions were more effective than those of shorter duration [66]. 

We did not specifically explore the impact of online delivery on treatment effects as this was only tested in one study; however, this delivery mode is becoming very popular and since our search was conducted, we are aware of some published and ongoing studies, with innovative approaches seeking to overcome issues with how to deliver depression prevention interventions on a large scale [65,67].

### 4.2. Strengths and Weakness of the Study

This study represents a secondary and post-hoc exploratory analysis of data from a larger Cochrane systematic review [12]. Additionally, the use of subgroup analyses to test differences in effectiveness based on various factors does not provide robust assurance of any true treatment differences given they represent indirect comparisons; head-to-head trials of different approaches are the only robust way to investigate the impact of different treatment approaches on outcomes. Given this, the results should be treated with some caution and used to derive hypotheses to be tested in further studies, for example, studies that incorporate a head-to-head design. Further caution in interpretation is required because of the limited power to adequately test effectiveness of approaches to intervention where there were very few trials. 

We are aware that in several instances, our results do not reflect the positive findings of trial authors in the original paper publications. It should be noted that the software used to undertake these meta-analyses [19] might implement analyses that are less sensitive than the analyses employed by trial authors. Additionally, we have used final scores, as this is primarily what is reported in the original publications, and these data provide a less sensitive measure of change than do change scores.

It also should be noted that the dataset for this study was drawn from a Cochrane review [12], the search for which was undertaken late in 2010, meaning that there are a number of relevant trials that have been published since that are not included in this analysis. 

### 4.3. Strengths and Weaknesses in Relation to other Studies, Discussing Important Differences in Results 

Several previous systematic reviews and meta-analyses have been undertaken, assessing the effectiveness of depression prevention programs generally and on the whole conclude that they are likely to be of benefit [7,8,10,11,15]. Only one of these reports a meta-analysis based on depressive diagnosis, and in line with our recent update of the Cochrane systematic review [12] shows a reduction in the number of diagnoses [15]. These two reviews note the small number of trials that have depressive diagnosis as an outcome and conclude that more studies are required to “prove the concept” [12,15], primarily because of this issue. 

However, few meta-analyses have attempted to identify which approaches to depression prevention may be more promising, nor formats suitable for large scale roll-out. Given findings of our updated Cochrane systematic review showing that both targeted and universal interventions do have ongoing effects (albeit only till 3 to 9 months for universal interventions), this would now seem a priority. Our study is the only we know of to explore whether the therapeutic approach or specific program type modifies the overall treatment effects of depression prevention programs for children and adolescents. Brunwasser [15] looked at one specific type of CBT intervention program, the Penn Resiliency Programs, and while showed some inconsistency in the findings, highlighted promising results. A meta-analysis of 69 programs to reduce depression in children, adolescents, adults and older adults highlighted the importance of delivering more than eight sessions that were of longer duration, delivery by health care professionals and of using multicomponent programs that included an element of competency training (e.g., skill training, social resistance skills) [66]. Our analysis showed that the number of sessions may be important but showed no impact of who delivered the programs. Differences may be due to there being far less variation in the type of therapeutic approaches used in the trials included in our review, where the majority were CBT-based programs. 

It may be that different approaches are more effective for targeted compared with universal interventions. We have shown that CBT, and for targeted interventions, the Coping with Stress Program in particular, hold some promise in terms of preventing depression in children and adolescents, although data on other approaches is very limited. It is important to note that a recent well designed effectiveness study did not find the Coping with Stress program delivered as a universal intervention prevented depression compared to no intervention (standard school curriculum) [64]. Indeed, the work by Stice and colleagues [58,59] shows a wide range of interventions were equally effective; and our Cochrane review [12] showed no difference between depression prevention programs and placebo or active comparison groups. This highlights the concern that there may be an important placebo effect operating. 

### 4.4. Implications

Preventing depressive disorder is a tantalizing goal. On the one hand many studies show promise, and indeed are mostly similar in content, and yet it has thus far proved impossible to get consistent results, or to take the programs to scale effectively. This does not mean that our efforts should stop, but we should be taking a new tack. 

New studies are constantly being published in this area; however, many are reiterations of past approaches. It is important to take stock of the large number of trials undertaken so far and stop repeating these. While similar in content, there are differences in the specific techniques included in each program as well as the approach to implementation in terms of the types of supporting materials and how these are used [10]. This is not dissimilar to the treatment field where substantial variations in treatment protocols for CBT in terms of the approaches and techniques they emphasise have been highlighted [68] with similar calls to explore the most effective components [68,69]. In the area of prevention, we now need to identify the components of the programs that might be the most effective and combine these using an effective mode of delivery that can be disseminated in a scalable strategy that ensures good uptake of the program. Many trials have good processes in place to try and ensure treatment fidelity, but in any ongoing delivery of a prevention program across a large population, it is going to be difficult to match these efforts and even with these in place there can be variability in effect within the same program. 

Perhaps the use of computerized delivery of interventions is a way forward. This field is in its infancy currently, but results of trials of computerized CBT indicate potential benefits for treating depression in adolescents [26,70]. It is certainly scalable and consistent but whether this mode of delivery is effective across large populations is not yet known. 

It is difficult to determine the active components of CBT programs that may have resulted in the treatment effects demonstrated, largely due to inconsistent reporting of the component skills and techniques used and the approach in terms of supporting materials. Our study points to the Penn Resiliency Program and Coping with Stress program as potential candidate for large scale roll out with both having a particular focus on cognitive restructuring (and problem solving in the Penn Resiliency Program); however, we have already noted the complicated screening process used in some of the most effective studies. This is not feasible in terms of a public health intervention. It is salutary that its effectiveness was not shown in a real world trial [64]. Similarly, particularly for studies that delivered PRP to universal interventions there was considerable variation across studies. 

Our findings are based on observational data from exploratory post-hoc subgroup analysis and hypotheses derived from this should be tested in a-priori head-to-head RCTs to test which approaches, programs or components of these should be implemented in large-scale public health initiatives. Such trials should measure the onset of depressive disorder as an outcome at 12-month follow-up or longer. Scalability should be tested, particularly in terms of implementation in a universal population, or identification of targeted populations and who and how content is delivered. Technology based approaches to delivery have appeal but like any other approach need to be rigorously tested. We have not yet found the Holy Grail of an effective prevention program but the quest should continue. 

## 5. Conclusions

Large numbers of trials testing the effectiveness of depression prevention interventions have been tested, and while overall they show promising results, there is considerable variability across study outcomes. It is important now to turn our attention to identifying the most effective approaches to depression prevention that are also scalable as large scale public health interventions. CBT certainly appears to be good contender, however, CBT includes a large range of skills, techniques and supporting materials that have been variously incorporated into treatment protocols to date, so that even within this approach it is still the case that the most effective core components need to be identified. Further, there may other approaches that might be effective; few studies have employed IPT, although this approach appears promising. More research is needed to identify the specific components of CBT and other therapies that are most effective, or indeed if there are other approaches that are more effective in reducing the risk of future depressive episodes. It is imperative that prevention programs are suitable for large-scale rollout, and that emerging popular modes of delivery, are considered. Unfortunately, to the question “Are we there yet?” the answer must be “No. We still have a way to go!”
